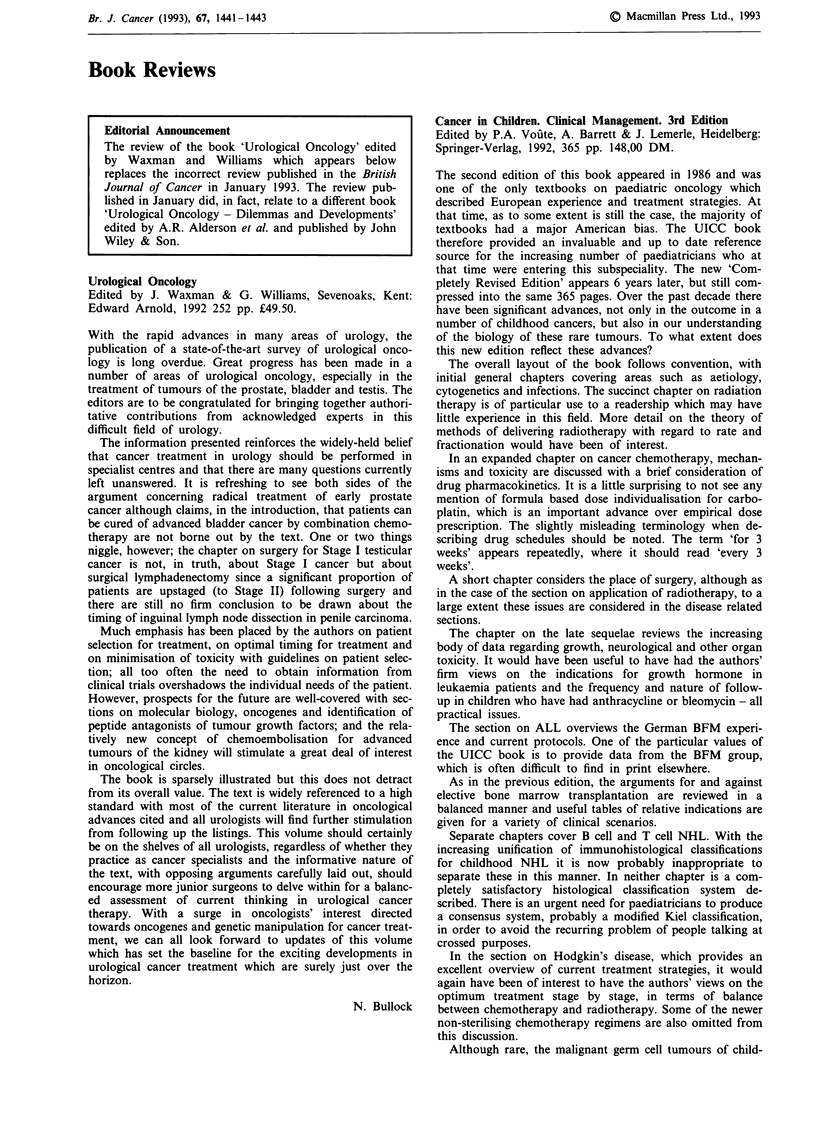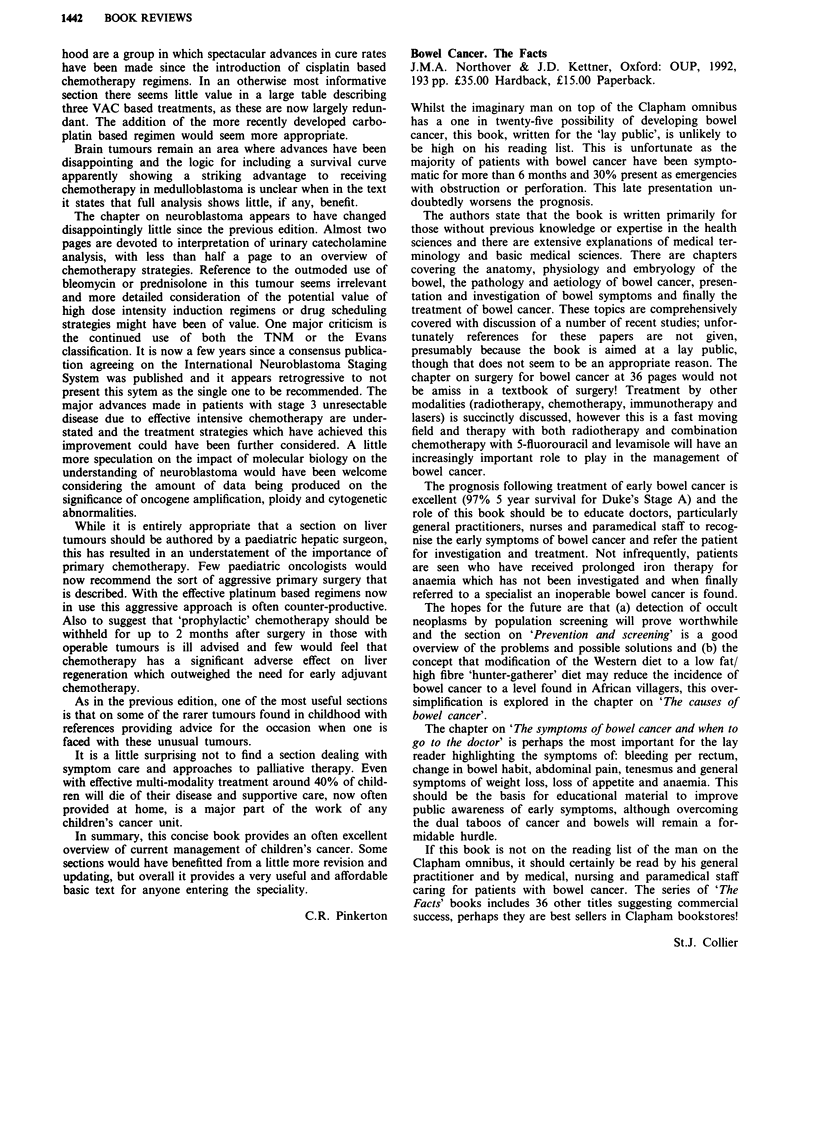# Cancer in Children. Clinical Management. 3rd Edition

**Published:** 1993-06

**Authors:** C.R. Pinkerton


**Cancer in Children. Clinical Management. 3rd Edition**
Edited by P.A. Voûte, A. Barrett & J. Lemerle, Heidelberg: Springer-Verlag, 1992, 365 pp. 148,00 DM.

The second edition of this book appeared in 1986 and was one of the only textbooks on paediatric oncology which described European experience and treatment strategies. At that time, as to some extent is still the case, the majority of textbooks had a major American bias. The UICC book therefore provided an invaluable and up to date reference source for the increasing number of paediatricians who at that time were entering this subspeciality. The new 'Completely Revised Edition' appears 6 years later, but still compressed into the same 365 pages. Over the past decade there have been significant advances, not only in the outcome in a number of childhood cancers, but also in our understanding of the biology of these rare tumours. To what extent does this new edition reflect these advances?

The overall layout of the book follows convention, with initial general chapters covering areas such as aetiology, cytogenetics and infections. The succinct chapter on radiation therapy is of particular use to a readership which may have little experience in this field. More detail on the theory of methods of delivering radiotherapy with regard to rate and fractionation would have been of interest.

In an expanded chapter on cancer chemotherapy, mechanisms and toxicity are discussed with a brief consideration of drug pharmacokinetics. It is a little surprising to not see any mention of formula based dose individualisation for carboplatin, which is an important advance over empirical dose prescription. The slightly misleading terminology when describing drug schedules should be noted. The term 'for 3 weeks' appears repeatedly, where it should read 'every 3 weeks'.

A short chapter considers the place of surgery, although as in the case of the section on application of radiotherapy, to a large extent these issues are considered in the disease related sections.

The chapter on the late sequelae reviews the increasing body of data regarding growth, neurological and other organ toxicity. It would have been useful to have had the authors' firm views on the indications for growth hormone in leukaemia patients and the frequency and nature of follow-up in children who have had anthracycline or bleomycin – all practical issues.

The section on ALL overviews the German BFM experience and current protocols. One of the particular values of the UICC book is to provide data from the BFM group, which is often difficult to find in print elsewhere.

As in the previous edition, the arguments for and against elective bone marrow transplantation are reviewed in a balanced manner and useful tables of relative indications are given for a variety of clinical scenarios.

Separate chapters cover B cell and T cell NHL. With the increasing unification of immunohistological classifications for childhood NHL it is now probably inappropriate to separate these in this manner. In neither chapter is a completely satisfactory histological classification system described. There is an urgent need for paediatricians to produce a consensus system, probably a modified Kiel classification, in order to avoid the recurring problem of people talking at crossed purposes.

In the section on Hodgkin's disease, which provides an excellent overview of current treatment strategies, it would again have been of interest to have the authors' views on the optimum treatment stage by stage, in terms of balance between chemotherapy and radiotherapy. Some of the newer non-sterilising chemotherapy regimens are also omitted from this discussion.

Although rare, the malignant germ cell tumours of childhood are a group in which spectacular advances in cure rates have been made since the introduction of cisplatin based chemotherapy regimens. In an otherwise most informative section there seems little value in a large table describing three VAC based treatments, as these are now largely redundant. The addition of the more recently developed carboplatin based regimen would seem more appropriate.

Brain tumours remain an area where advances have been disappointing and the logic for including a survival curve apparently showing a striking advantage to receiving chemotherapy in medulloblastoma is unclear when in the text it states that full analysis shows little, if any, benefit.

The chapter on neuroblastoma appears to have changed disappointingly little since the previous edition. Almost two pages are devoted to interpretation of urinary catecholamine analysis, with less than half a page to an overview of chemotherapy strategies. Reference to the outmoded use of bleomycin or prednisolone in this tumour seems irrelevant and more detailed consideration of the potential value of high dose intensity induction regimens or drug scheduling strategies might have been of value. One major criticism is the continued use of both the TNM or the Evans classification. It is now a few years since a consensus publication agreeing on the International Neuroblastoma Staging System was published and it appears retrogressive to not present this sytem as the single one to be recommended. The major advances made in patients with stage 3 unresectable disease due to effective intensive chemotherapy are understated and the treatment strategies which have achieved this improvement could have been further considered. A little more speculation on the impact of molecular biology on the understanding of neuroblastoma would have been welcome considering the amount of data being produced on the significance of oncogene amplification, ploidy and cytogenetic abnormalities.

While it is entirely appropriate that a section on liver tumours should be authored by a paediatric hepatic surgeon, this has resulted in an understatement of the importance of primary chemotherapy. Few paediatric oncologists would now recommend the sort of aggressive primary surgery that is described. With the effective platinum based regimens now in use this aggressive approach is often counter-productive. Also to suggest that 'prophylactic' chemotherapy should be withheld for up to 2 months after surgery in those with operable tumours is ill advised and few would feel that chemotherapy has a significant adverse effect on liver regeneration which outweighed the need for early adjuvant chemotherapy.

As in the previous edition, one of the most useful sections is that on some of the rarer tumours found in childhood with references providing advice for the occasion when one is faced with these unusual tumours.

It is a little surprising not to find a section dealing with symptom care and approaches to palliative therapy. Even with effective multi-modality treatment around 40% of children will die of their disease and supportive care, now often provided at home, is a major part of the work of any children's cancer unit.

In summary, this concise book provides an often excellent overview of current management of children's cancer. Some sections would have benefitted from a little more revision and updating, but overall it provides a very useful and affordable basic text for anyone entering the speciality.

C.R. Pinkerton